# Decreased resistance to bacterial cold-water disease and excessive inflammatory response in ayu (*Plecoglossus altivelis)* reared at high water temperature

**DOI:** 10.3389/fimmu.2023.1101491

**Published:** 2023-02-02

**Authors:** Goshi Kato, Hayato Nakajima, Kyuma Suzuki, Yuhei Kanzawa, Chihaya Nakayasu, Kosei Taguchi, Osamu Kurata, Motohiko Sano

**Affiliations:** ^1^ Laboratory of Fish Pathology, The Marine Faculty of Bioresources Phylum, Tokyo University of Marine Science and Technology, Tokyo, Japan; ^2^ Gunma Prefectural Fisheries Experiment Station, Gunma, Japan; ^3^ Nansei Field Station, Fisheries Technology Institute, Japan Fisheries Research and Education Agency, Mie, Japan; ^4^ Laboratory of Aquatic Medicine, School of Veterinary Medicine, Faculty of Veterinary Medical Science, Nippon Veterinary and Life Science University, Tokyo, Japan

**Keywords:** Flavobacterium psychrophilum, poikilotherm, global warming, aquaculture, seedling production, vaccination

## Abstract

**Introduction:**

Temporal elevation of water temperature positively affects immune activity and disease resistance in poikilothermic teleost fish. The ayu, *Plecoglossus altivelis*, an important fish species for Japanese freshwater fisheries, is usually produced under higher water temperatures than the natural conditions to facilitate rapid growth. However, it has been reported that rearing fish at higher water temperatures inhibits the development of the thymus, suggesting that resistance to infectious diseases is reduced in ayu reared at higher water temperatures. Here, we show that decreased resistance to bacterial cold-water disease and excessive inflammatory responses occurred in ayu reared at 22°C compared with those reared at lower temperatures.

**Methods:**

Ayu larvae were reared at 12°C, 15°C and 22°C for 77 days and fed 3% of their body weight. Thymus index and condition factor was calculated after the fish rearing. Then, ayu reared at the different temperatures were challenged with Flavobacterium psychrophilum and the fish were sampled for histopathology and gene expression analyses. Further, the fish were vaccinated with formalin-killed F. psychrophilum and continuously reared at the three different water temperatures. Serum antibody titer was determined by ELISA and cumulative mortality in each group was recorded after the bacterial challenge.

**Results:**

Ayu reared at 22°C showed a significantly lower thymus index and higher condition factor than those reared at lower temperatures. Infiltrated leukocytes and many melanin pigments were frequently observed in the adipose tissues and spleens of ayu reared at 22°C, respectively, but not in those reared at 12°C. The gene expression levels of inflammatory cytokines such as IL-1β, IL-8 and TNFα in the spleen were significantly higher in the 22°C group than in the 12°C group. The cumulative survival rate after challenge with *Flavobacterium psychrophilum* was 51.7%, 40.0% and 13.3% in the 12°C, 15°C and 22°C groups, respectively. The relative percent survival values of vaccinated fish reared at 15°C and 22°C groups were lower than those reared at 12°C. Moreover, the specific antibody titer of the vaccinated fish was the lowest in the 22°C group and the highest in the 12°C group.

**Discussion:**

These results suggest that rearing the fish under high water temperature causes excessive inflammatory responses similar to metabolic inflammation in human obesity, resulting in a decrease of disease resistance. In addition, thymic involution induced by higher water temperature probably leads the poor response to vaccination. The present study provides insights into the physiological and immunological changes of fish under global warming.

## 1 Introduction

Elevation of body temperature is an excellent strategy for biological defense against pathogens in homeothermic animals. Fever mechanistically and functionally regulates inflammation *via* NF-κB signaling pathway and induction of inflammatory macrophages by TNFα production during infections (1, 2). Unlike mammals, body temperature of poikilotherms such as teleost fish is dependent on the environmental temperature. These animals behaviourally raise the body temperature by seeking warmer temperature when they are infected with pathogens. High water temperature treatment is usually used to cure of infectious diseases in aquaculture (3, 4). In fact, antibody production is facilitated under high water temperatures, but the peak response was delayed as the temperature decreased in numerous fish species (5). In this way, temporal elevation of water (body) temperatures results in increase level of immune activity and disease resistance in teleost fish. However, little is known how the continuous rise in water temperature, e.g., global warming, affect immune activity and disease resistance in teleost fish.

The ayu (*Plecoglossus altivelis*) is an amphidromous fish species that has a 1-year lifespan. Wild ayu spawn in the middle or lower reaches of rivers during the autumn season, and the larvae hatches and immediately migrate to the coastal area of the sea. The larvae (less than 48 mm body length) spend the winter season in brackish water around estuaries, where the water temperature is between 5°C and 10°C (6). Then, the juvenile fish (body length = 50-90 mm) migrate to the upper reaches after the water temperature rises to 10°C-13°C in the following spring (6, 7). The fish is the most important species for Japanese freshwater fisheries. The output amounts one-third of the total value of freshwater fishery and aquaculture production (http://www.e-stat.go.jp “Accessed 8 Oct 2022”). Ayu is also cultured not only for food purposes but also for release into the river water system as popular game fishing targets, therefore, many local fisheries stations produce ayu for aquaculture and release. The methods to produce ayu larvae have been summarized in “The manual for ayu seedlings production” edited by National Lake River Aquaculture Study Group and Ayu Initial Feed Research Group in 1999 (only available in Japanese). After artificial spawning in the autumn season, hatched larvae are reared in freshwater ponds (hatchery and nursery period). The larvae are transferred to seawater or artificial seawater (3–5 ppm) and reared for 70–100 days (larval period). The larvae (0.1–1.0 g) are transferred again to indoor freshwater ponds until shipment (larval-juvenile period). We previously surveyed water temperatures during ayu production at several local fisheries stations in Japan (8). In the larval period and larval-juvenile period, the rearing water is usually warmed by an oil boiler, or the fish are reared in the indoor pool to keep the temperature higher (15°C-22°C) than the natural conditions (5°C-13°C). Ito et al. (9) reported that 13°C–15°C was suitable for ayu larval production, while a water temperature under 11°C led to a decrease in the food intake, growth rate and yield rate of the fish (9). Sakano and Uchino (2011) demonstrated that water temperature at 20°C and 25°C was suitable for the growth of wild ayu larvae caught in brackish water, the larval fish reared at 15°C showed the lower growth rate than those reared at the above temperatures (10). The food intake in teleost fish increase as a result of activated feeding behavior, swimming rate and metabolic rate in high water temperatures, resulting high growth rate and yield rate (11). Thus, cultured ayu are usually produced under higher water temperatures, while wild ayu experience low temperatures (less than 10°C) under natural conditions. As a result, cultured fish that are larger than those grown in natural conditions are supplied at the beginning of June when ayu fishing restrictions are lifted.

Miwa et al. (12) reported that the relative thymus volume (= thymus volume [mm^3^]/body length [cm]) of cultured ayu was significantly smaller than that of wild ayu captured in Lake Biwa, Shiga, Japan (12). Furthermore, they showed that rearing the fish at a high water temperature (17.2°C) resulted in impairment of thymus development, while rearing of ayu at a low water temperature (13.8°C) resulted in a normal thymus volume (12). The influence of water temperature on thymus development was further confirmed by Hara et al. (16), who found that the relative thymus volume of ayu reared at 20°C was significantly smaller than that of ayu reared at 15°C (13). The low relative thymus volumes of ayu reared at high water temperature (18°C and 20°C) were maintained when the fish were transferred and further reared in another tank with low water temperature (15°C) (14). The thymus is the hematopoietic tissue that produces mature T lymphocytes, which play critical roles in the acquired immune response. Thus, impairment of the thymus may cause a decrease in resistance to infectious pathogens.

Bacterial cold-water disease (BCWD) caused by *Flavobacterium psychrophilum* makes severe losses of cultured and released ayu in the river water system in Japan (15–17). In this study, we investigated the effect of continuous rise in water temperature to resistance and immune response against BCWD using ayu produced under different water temperatures. Present data suggest that the current protocol for ayu production is needed to improve, especially in terms of water temperature, and provides insights into the effect of global warming to physiological and immunological functions in poikilothermic teleost fish.

## 2 Materials and methods

### 2.1 Fish rearing

Ayu (*P. altivelis*) from an amphidromous stock were used in this study. The fish stock, SE8, was derived from wild fish collected from an upstream migrating population in the Edogawa River and was maintained for eight generations at the Gunma Prefectural Fisheries Experiment Station. The fish rearing schedules are shown in **Supplementary Figure 1**. Fish (average body weight = 0.5 g) were reared in 1-ton tanks with flow-through water conditions at 12°C, 15°C or 22°C for 77 days (from January 16 to April 3, 2019) and then acclimatized at 15°C for 7 days. Fish were sampled at random, and the body length and body weight were recorded on April 9, 2019. These fish were also used for calculation of the thymus index (during acclimatization), a challenge test, gene expression analyses and histopathological examination as described below (Experiment 1, **Supplementary Figure 1A**). In the same way, fish reared at 12°C, 15°C or 22°C for 85 days (from January 16 to April 11, 2019) were subjected to vaccination (Experiment 2, **Supplementary Figure 1B**). Vaccinated fish were further reared at the same water temperature for 21 days, acclimatized at 15°C for 7 days and used for thymus index calculation, antibody titration and a challenge test. The fish were fed every day with standard fish pellets at a rate of 3% of fish body weight. The fish weight and body length were measured after anesthetization with FA 100 (final concentration = 20 ppm, DS Pharma Animal Health, Osaka, Japan). All animal experiments were approved by the Committee of Animal Experiments at Tokyo University of Marine Science and Technology (TUMSAT) and performed in accordance with the “Guidelines for the Care and Use of Laboratory Animals” of TUMSAT under the international guideline “Act on Welfare and Management of Animals” (Ministry of the Environment of Japan).

### 2.2 Thymus index

Thymus volume was measured using computed tomography (CT) scanning following the protocol described in Takada et al. (14) with some modifications. The heads of ayu reared under different temperatures (*n* = 4–5) were fixed in Davidson’s solution (30% ethanol, 10% formalin, 10% acetic acid). The fixed samples were incubated in 2.5% phosphomolybdic acid for 6 days under dark conditions, washed three times with distilled water and subjected to CT scanning using an X-ray CT system for laboratory animals (Latheta LCT-100A, Hitachi Ltd.) with a slice thickness of 0.1 mm. The thymus volume was determined using the image analysis software Horos v 2.2.0, and the thymus index was calculated as follows: thymus index = [thymus volume (cm^3^)/body weight (g)] × 100.

### 2.3 Challenge test

*Flavobacterium psychrophilum* strain GMA 0330 (18) isolated from diseased ayu was cultured in modified Cytophaga (MCY) broth at 15°C for 24 h (19). The bacterial cultures were serially diluted with MCY broth and incubated on MCY agar at 15°C for 72 h to count colony forming units (CFU). Ayu reared at the different temperatures were intraperitoneally injected with 5.6 × 10^5^ CFU/fish of the bacteria and kept in three different tanks for each group. A group without challenge was also prepared as a control. The mortalities were recorded for 12 days after the challenge Kaplan‒Meier survival curves were generated. Significant differences (*p* < 0.05) in the cumulative survival rate were detected using one-way ANOVA and further analyzed with Ryan’s *post hoc* test.

### 2.4 Histopathology and immunofluorescence assay

The spleens of ayu reared under different temperatures were sampled prior to the challenge (0 h), at 24 h and 48 h after the challenge. The fish bodies were also sampled *via* round slices taken from between the end of the dorsal fin and adipose fin, including the intestine and trunk kidney, prior to the challenge. Each tissue sample was fixed with Davidson’s solution, decalcified in phosphate-buffered saline containing 0.3 M EDTA for 4 days, embedded in paraffin and sectioned at 3-*µ*m thickness. The pathological changes in the spleen were observed under an ECLIPSE Ci digital light microscope (Nikon, Tokyo, Japan) after hematoxylin and eosin (HE) staining.

For the immunofluorescence assay, the spleen sections were blocked with 1% skim milk diluted in Tris-buffered saline (TBS) for 1 h at 4°C and incubated with rabbit serum raised against *F. psychrophilum* (1:200) at 4°C for 1 h. After washing three times with TBS, the sections were incubated with goat anti-rabbit IgG Alexa Fluor 488 conjugate (Abcam, Cambridge, UK) diluted to 1:500 at 4°C for 1 h. The sections were counterstained with Hoechst 33342 (Thermo Fisher Scientific, MA, USA) and mounted with ProLong Gold Antifade Mountant (Thermo Fisher Scientific). Digital images were captured and analyzed with a fluorescence microscope and NIS Elements software (Nikon, Tokyo, Japan).

### 2.5 Gene expression analysis

A piece of each spleen sample collected at 0 h and 24 h after the challenge was also stored in nucleic acid preservation buffer (20) at –80°C until use. Total RNA was extracted from the spleen sample using RNAiso Reagent (TAKARA, Tokyo, Japan) following the manufacturer’s instructions. First-strand cDNA was synthesized with 500 ng of total RNA using MMLV reverse transcriptase (Thermo Fisher Scientific) following the manufacturer’s instructions. The gene expression levels of CD4-1, CD8α, interleukin (IL)-1β, IL-8, IL-10 and TNFα were investigated using quantitative RT-PCR (qPCR). The primers used in qPCR were designed using Primer3Plus software (http://primer3plus.com, accessed on October 9, 2022) and are shown in **Supplementary Table 1**. The reaction mixtures containing each cDNA sample were prepared using THUNDERBIRD SYBR qPCR Mix (Toyobo, Tokyo, Japan) following the manufacturer’s instructions. qPCR was performed using a LightCycler 480 II (Roche Diagnostics, Manheim, Germany) following the manufacturer’s instructions. The gene expression level was calculated from a standard curve for each gene, and the EF1α level was used as an internal control. The gene expression values were taken as the mean values (*n* = 4-5) and presented as the fold-change relative to the value of each gene at 0 h after the challenge in the 12°C-reared group, which was set to 1.0. Significant differences (*P* < 0.05) in the gene expression levels were detected using two-way ANOVA and further analyzed with Ryan’s *post hoc* test (among the experimental groups) or Student’s *t* test (between 0 h and 24 h after the challenge in each group).

### 2.6 Vaccination and challenge test

*Flavobacterium psychrophilum* strain GMA 0330 was cultured in MCY broth as above and inactivated by adding formalin (final concentration of 0.3% v/v). Inactivation was confirmed by incubating the culture medium on MCY agar at 15°C for 48 h. Ayu reared at 12°C, 15°C and 22°C were injected with formalin-killed *F. psychrophilum* cells (FKCs, 6.7 × 10^7^ CFU/fish). Ayu injected with phosphate-buffered saline (PBS) were used as negative controls for the experiment. The experimental fish were challenged with *F. psychrophilum* strain GMA 0330 (5.6 × 10^6^ CFU/fish) at 4 weeks post-vaccination, and mortality was recorded for 12 days after the challenge. The relative percent survival (RPS) was calculated as follows: (cumulative mortality of PBS-injected fish/cumulative mortality of the vaccinated fish) × 100. Kaplan‒Meier survival curves were generated, and the log-rank test was used to detect a significant difference between the survival curve of vaccinated fish and that of PBS-injected fish in each temperature group.

### 2.7 ELISA

Blood was removed from the vaccinated fish at 4 weeks post-vaccination by venipuncture and allowed to clot at 4°C overnight, and the serum was separated by centrifugation at 3,000 ×*g* for 10 min. The serum was kept at –80°C until use. *F. psychrophilum* cultured in MCY broth (OD 660 nm = 0.298) was sonically disrupted for 5 min (a 5 s sonication and a 1 sec cooling on ice, 30% amplitude) using a SONICS VCX-130 (Sonics & Materials, CT, USA). The antigen solution was diluted twofold with 50 mM carbonate buffer (pH = 9.6), added to each well of a 96-well plate (ELISA Plate H, Sumitomo Bakelite, Tokyo, Japan), and incubated overnight at 4°C. After removing the antigen solution, the 96-well plate was blocked with 1% skimmed milk in Tris-buffered saline containing 0.5% Tween 20 (TBST) for 1 h at 4°C. After washing three times with TBST, the serum samples diluted 1:20 with the blocking solution were added to wells and incubated at 4°C for 1 h. The 96-well plate was washed three times with TBST and incubated with a 1:50 dilution of a monoclonal antibody against ayu serum Igs (21) in the blocking solution at 4°C for 1 h. Following three washes with TBST, the 96-well plate was incubated with goat anti-mouse IgG H&L conjugated with HRP (Abcam, Cambridge, UK) diluted 1:1,000 with the blocking solution. The plate was washed as well and incubated with SureBlue TMB 1-Component Microwell Peroxidase Substrate (KPL, MD, USA) for 1.5 min at room temperature following the manufacturer’s instructions. Finally, 1 N HCl was added to each well to stop the reaction, and the absorbance (450 nm) was measured using an MPR-A4i II (TOSO, Tokyo, Japan). The significance of differences (*p* < 0.05) was detected using two-way ANOVA (n = 5-10) and further analyzed with Ryan’s *post hoc* test (among the experimental groups) or Welch’s t test (between the PBS- and FKC-injected fish in each group).

## 3 Results

### 3.1 Condition factor and thymus index

The body length, body weight, condition factor and thymus index values of ayu reared under the different water temperatures are shown in **Table 1**. The mean values of body length and body weight were similar between the 12°C and 15°C groups, while the values in the 22°C group were significantly lower than those in the other groups. In contrast, the condition factor was the highest in the 22°C group and comparable between the 12°C and 15°C groups. As previously reported by Miwa et al. (12), Hara et al. (2005) (13) and Takada et al. (14), the thymus index was highest in the 12°C group, moderate in the 15°C group and lowest in the 22°C group.

**Table 1:** Body length, body weight, condition factor and thymus index values of ayu reared under different water temperatures.

Group	DTC^1^	BL (cm) ^2^	BW (g) ^3^	CF^4^	TI^5^
Ex. 1					
12°C	77	6.4 ± 0.2 ^a^	2.8 ± 0.3 ^a^	10.9 ± 0.6 ^b^	30.1 ± 6.2 ^a^
15°C	77	6.1 ± 0.3 ^b^	2.5 ± 0.4 ^b^	10.9 ± 0.6 ^b^	24.7 ± 8.1 ^ab*^
22°C	77	5.5 ± 0.3 ^c^	2.0 ± 0.3 ^c^	12.4 ± 0.5 ^a^	10.2 ± 13.1 ^b^
Ex. 2					
12°C	105	6.7 ± 0.4 ^a^	3.3 ± 0.7 ^a^	11.0 ± 1.5 ^b^	20.7 ± 4.0 ^a^
15°C	105	6.7 ± 0.4 ^a^	3.2 ± 0.6 ^a^	10.8 ± 0.7 ^b^	10.2 ± 2.7 ^b^
22°C	105	5.8 ± 0.5 ^b^	2.5 ± 0.5 ^b^	12.7 ± 0.9 ^a^	3.9 ± 1.5 ^c^

^1^ Days for temperature control.

^2^ Body length (*n* =10).

^3^ Body weight (*n* =10).

^4^ Condition factor: BW/BL^3^ × 1000 (*n* =10).

^5^ Thymus index: Thymus volume (mm^3^)/BW × 100 (n = 5, *n = 4).

Different letters show significant differences.

### 3.2 Challenge test

**Figure 1:**
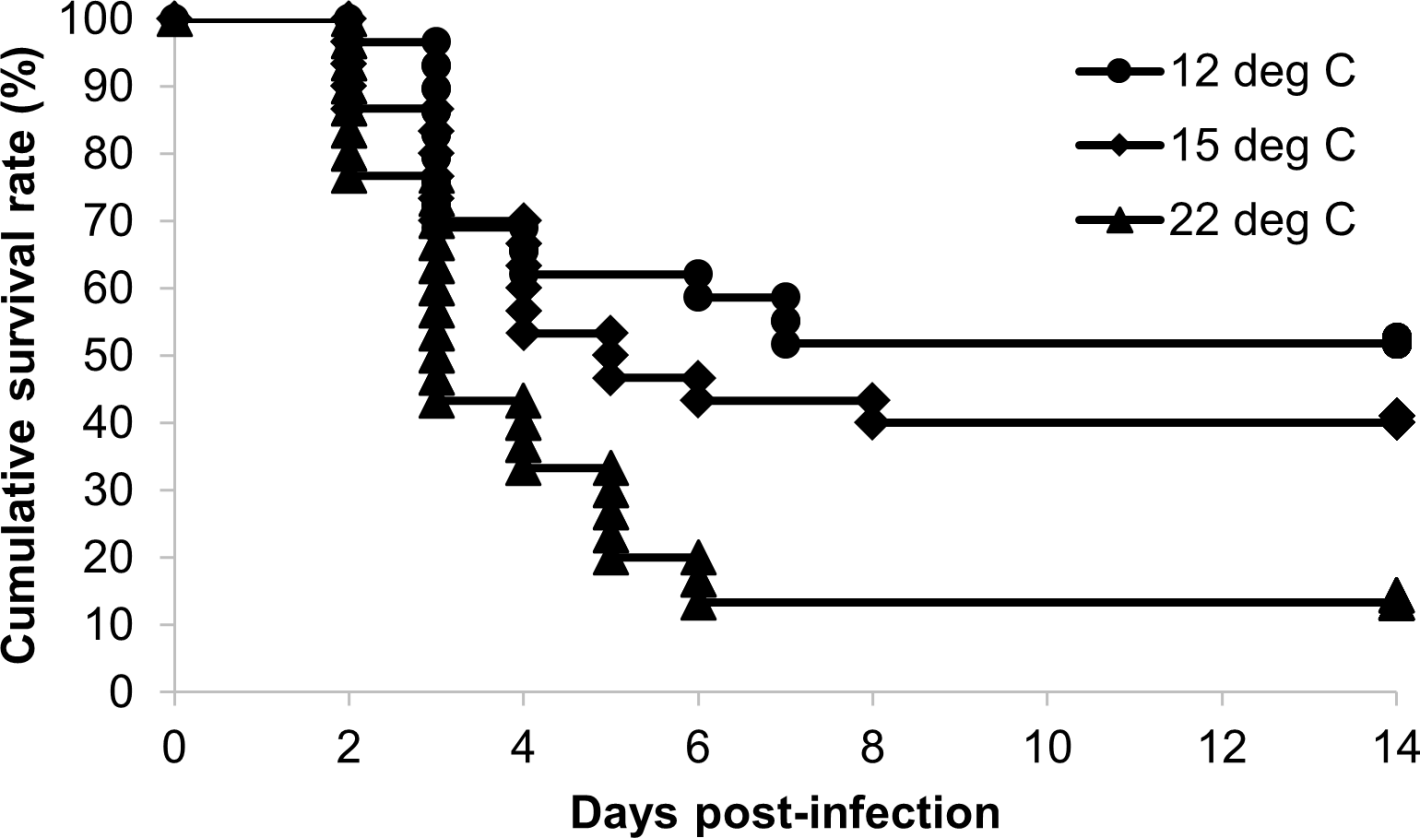
Kaplan‒Meier survival curves for ayu reared at different water temperatures after *Flavobacterium psychrophilum* challenge. Circles, diamonds and triangles indicate the cumulative survival rates of the 12°C (*n* = 29), 15°C (*n* = 30) and 22°C groups (*n* = 30), respectively.

Mortality was first observed at 2 days post-infection in all experimental groups (**Figure 1**), and the dead fish showed the typical symptoms of bacterial cold-water disease. The cumulative survival rates at 14 days post-infection were 51.7%, 40.0% and 13.3% in the 12°C group, 15°C group and 22°C group, respectively (**Table 2**). The survival rate in the 12°C group was significantly higher than those in the 15°C group and 22°C group (*P* < 0.05).

**Table 2:** Survival rates of ayu reared at different temperatures after *Flavobacterium psychrophilum* challenge.

Group	Survival rate (survived/total)
12°C 15°C 22°C	51.7%^a^ (15/29) 40.0%^b^ (12/30) 13.3%^b^ (4/30)

Different letters show significant differences.

### 3.3 Histopathology and immunofluorescence assay

**Figure 2:**
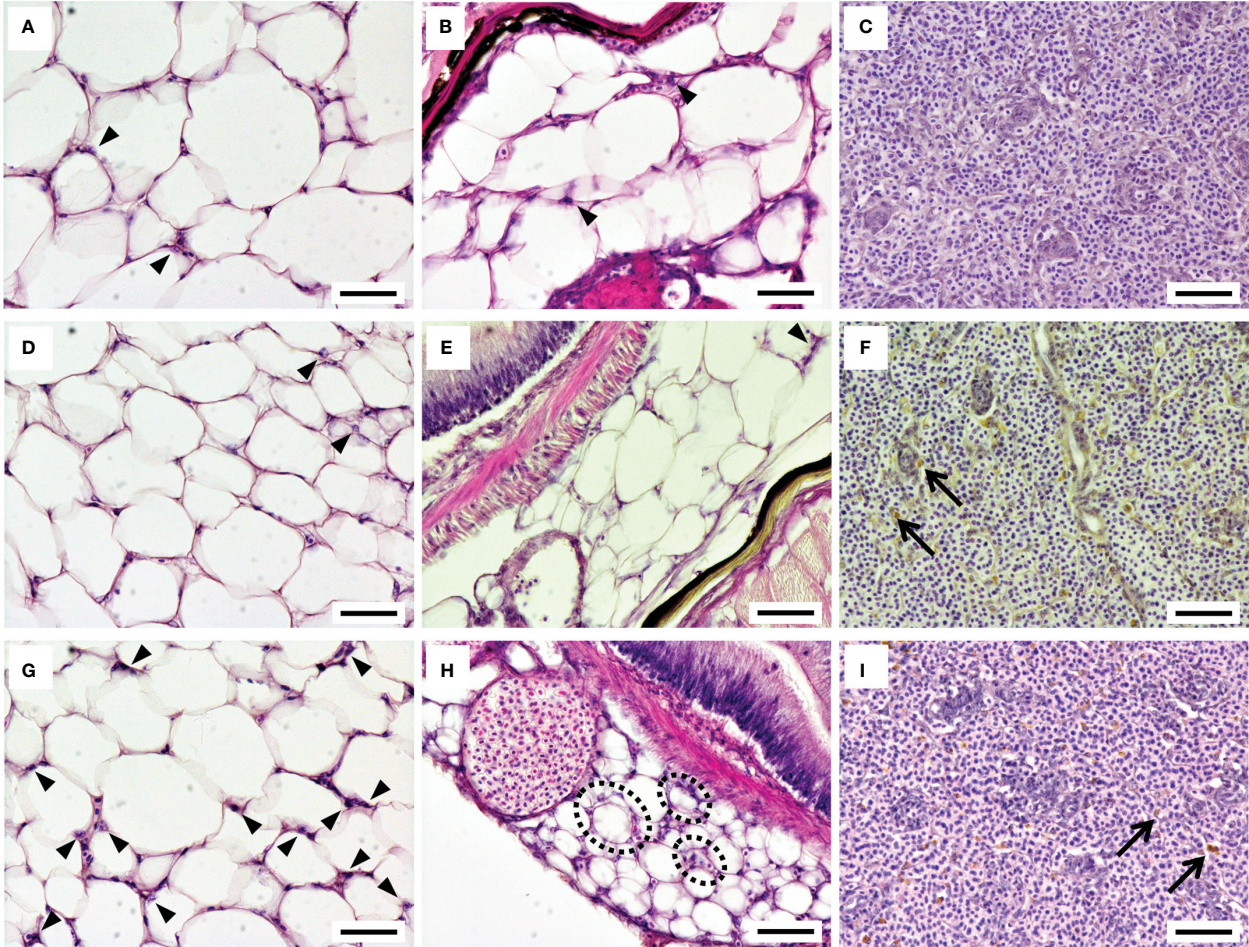
Histological features of the adipose tissue around the intestinal tract and the spleen in ayu reared at 12°C **(A-C)**, 15°C **(D-F)** and 22°C **(G-I)**. The left, middle and right panels show the adipose tissue around the intestinal tract on the upper (trunk kidney) side, on the lower (anal fin) side and in the spleen, respectively. Arrowheads and arrows indicate infiltrated leukocytes in the adipose tissue and melanin pigments in the spleen, respectively. Black dotted circles represent adipocytes surrounded by infiltrated leukocytes, producing structures similar to mammalian crown-like structures. Bar = 50 µm. Data are representative of three individuals in each group.

The adipose tissue around the intestinal tract in the upper (trunk kidney side) and lower (anal fin side) peritoneal cavity was observed under light microscopy after HE staining (**Figure 2**). A few leukocytes infiltrated into the adipose tissue in the 12°C group and 15°C group (**Figures 2A, B, D, E**), while a considerable number of migrated leukocytes were observed in the adipose tissue of the 22°C group (**Figures 2G, H**). Adipocytes surrounded by migrated leukocytes, which resembled crown-like structures in mammals, were often found in the 22°C group, especially in the adipose tissue at the lower side of the intestinal cavity (**Figure 2H**). Although the highest condition factor was recorded in the 22°C group, there was no remarkable difference in the size of adipocytes among the experimental groups. The spleens of ayu reared at 12°C showed normal histological features, while many melano-macrophages were observed in the spleens of ayu reared at 15°C and 22°C (**Figures 2C, F, I**).

**Figure 3:**
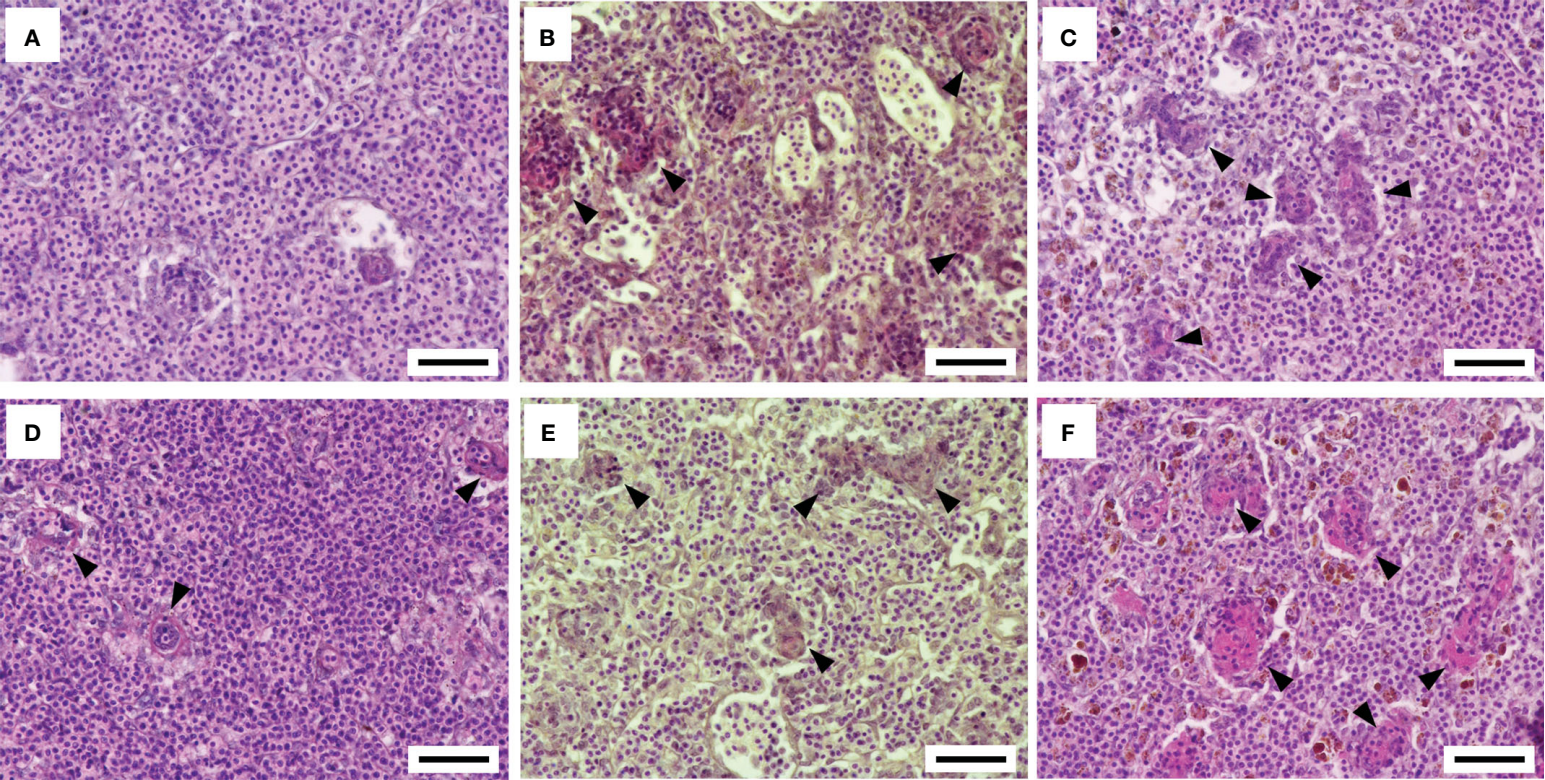
Histopathological features of the spleen after the *Flavobacterium psychrophilum* challenge in ayu reared at different water temperatures. **(A-C)** Spleen sections of ayu reared at 12°C **(A)**, 15°C **(B)** and 22°C **(C)** at 1 day post-infection. **(D-F)** Spleen sections of ayu reared at 12°C **(D)**, 15°C **(E)** and 22°C **(F)** at 2 days post-infection. Arrowheads indicate the regressive change in the ellipsoidal tissue. Bar = 50 µm. Data are representative of three individuals in each group.

**Figure 4:**
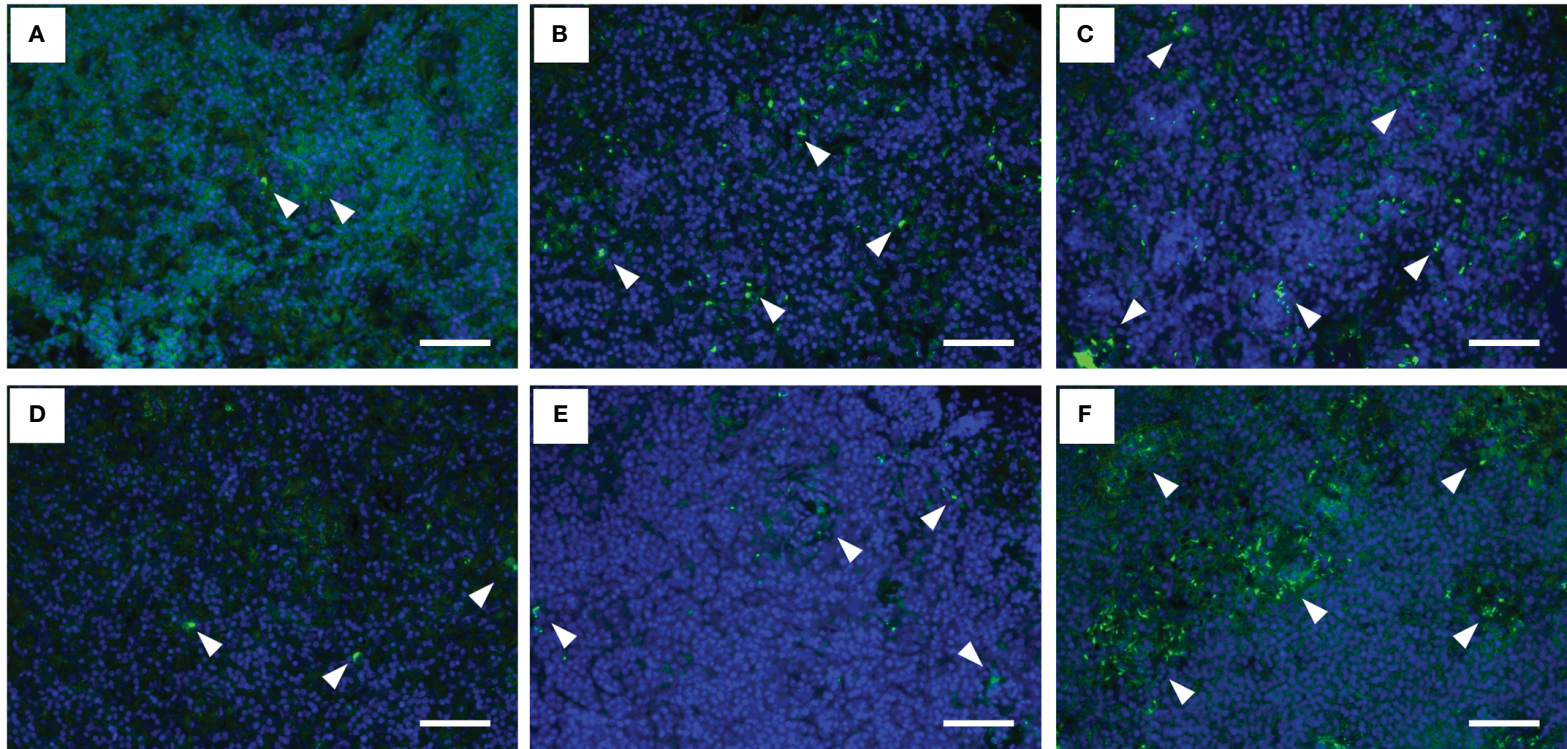
Fluorescence immunohistochemistry for *Flavobacterium psychrophilum* in the spleens of infected ayu reared at different water temperatures. **(A-C)** Spleen sections of ayu reared at 12°C **(A)**, 15°C **(B)** and 22°C **(C)** at 1 day post-infection. **(D-F)** Spleen sections of ayu reared at 12°C **(D)**, 15°C **(E)** and 22°C **(F)** at 2 days post-infection. Arrowheads indicate stained bacteria. Bar = 50 µm. Data are representative of three individuals in each group.

The histopathological features of the spleen after the *F. psychrophilum* challenge are shown in **Figure 3**. No remarkable changes were found in the spleen of the 12°C group at 24 h after the challenge (**Figure 3A**). Regressive changes such as pyknosis and degeneration in the ellipsoidal tissues appeared in the spleens of the 15°C and 22°C groups at 24 h after the challenge (**Figures 3B, C**). These pathological changes were also seen in the spleens of all groups at 48 h after the challenge (**Figures 3D-F**). Fluorescence signals for *F. psychrophilum* were detected around the ellipsoidal tissues of all challenged fish at 24 and 48 h after the challenge (**Figure 4**) but not in the spleens of ayu sampled prior to the infection (**Supplementary Figure 2**). Consistent with the histopathological changes, several bacterial signals were found in the spleens of the 12°C group, while the numbers of signals were clearly higher in the 15°C and 22°C groups at 24 h after the challenge (**Figures 4A-C**). Many signals were still observed in the 22°C group, but a few bacteria were detected in the spleens of the 12°C and 15°C groups 48 h after the challenge (**Figures 4D-F**).

### 3.4 Gene expression analysis

**Figure 5:**
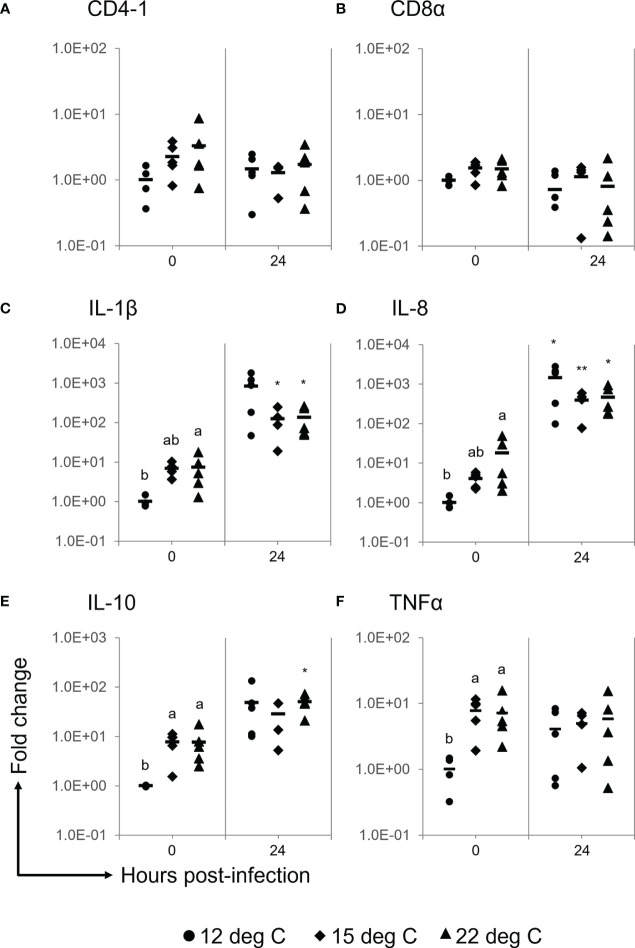
Gene expression analyses for CD4-1 **(A)**, CD8α **(B)**, IL-1β **(C)**, IL-8 **(D)**, IL-10 **(E)** and TNFα **(F)** in the spleens of ayu reared at different temperatures after *Flavobacterium psychrophilum* challenge. Black circles, diamonds and triangles represent individual data points of the 12°C group, 15°C group and 22°C group, respectively. The horizontal line indicates the mean value in the group (*n* = 4, 5). Different characters indicate significant differences among the three groups at the same time point as detected by ANOVA and the *post hoc* test. Asterisks indicate significant differences between 0 h and 24 h post-infection in the same experimental group as detected by Student’s t test (*, *P* < 0.05; ** *P* < 0.01).

There were no significant differences in the gene expression levels of CD4-1 and CD8α among either the experimental groups or the experimental periods (**Figures 5A, B**). The gene expression levels of IL-1β and IL-8 were significantly higher in the 22°C group than in the 12°C group prior to the challenge (**Figures 5C, D**). A significant difference was not observed in some cases, and these two inflammatory cytokine genes were upregulated at 24 h after the challenge in the spleens of each experimental group (**Figures 5C, D**). The IL-10 gene expression level prior to the challenge was significantly higher in the spleens of the 15°C and 22°C groups than in those of the 12°C group and then tended to be upregulated in all experimental groups after the challenge (**Figure 5E**). The TNFα gene was not upregulated after the challenge, while the gene expression level prior to the challenge was significantly higher in the 15°C and 22°C groups than in the 12°C group (**Figure 5F**).

### 3.5 Vaccination, challenge test and ELISA

**Figure 6:**
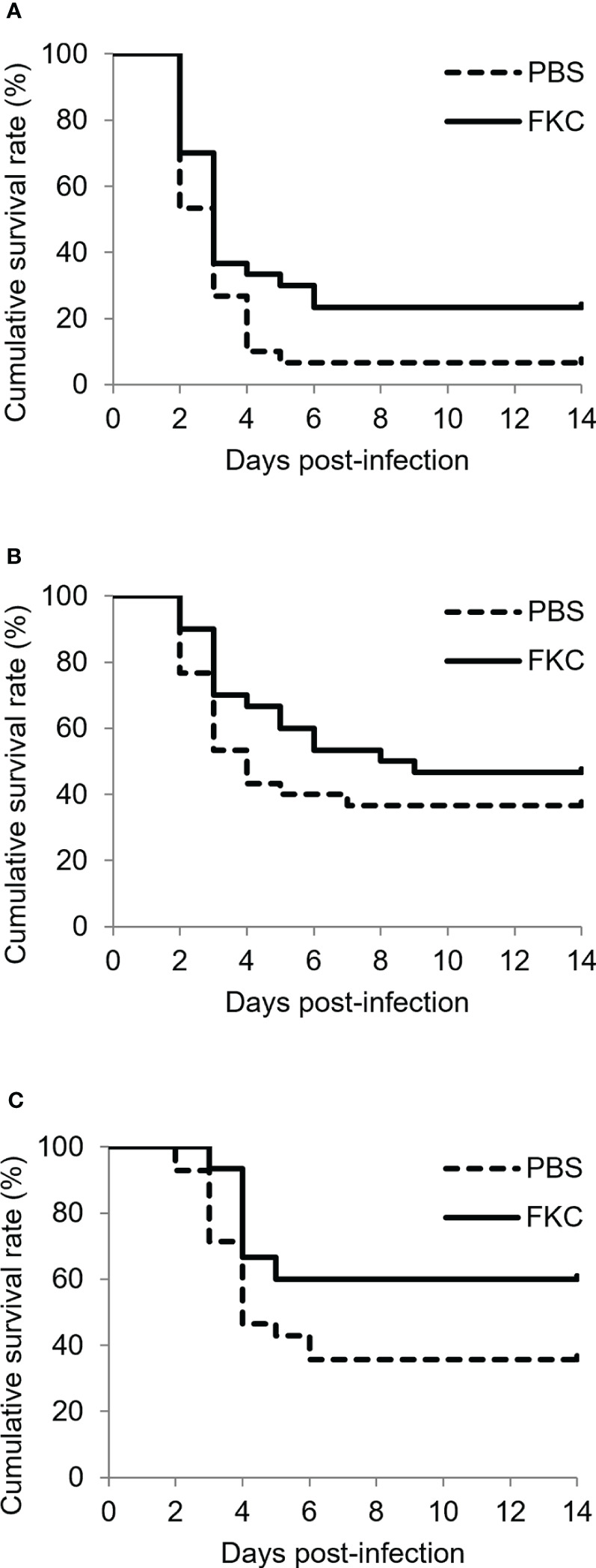
Kaplan‒Meier survival curves (*n* = 28-31) for FKC-vaccinated fish and PBS-injected fish in the 12°C group **(A)**, 15°C group **(B)** and 22°C group **(C)** after *Flavobacterium psychrophilum* challenge. Solid lines and dotted lines indicate FKC-vaccinated fish and PBS-injected fish, respectively.

**Figure 7:**
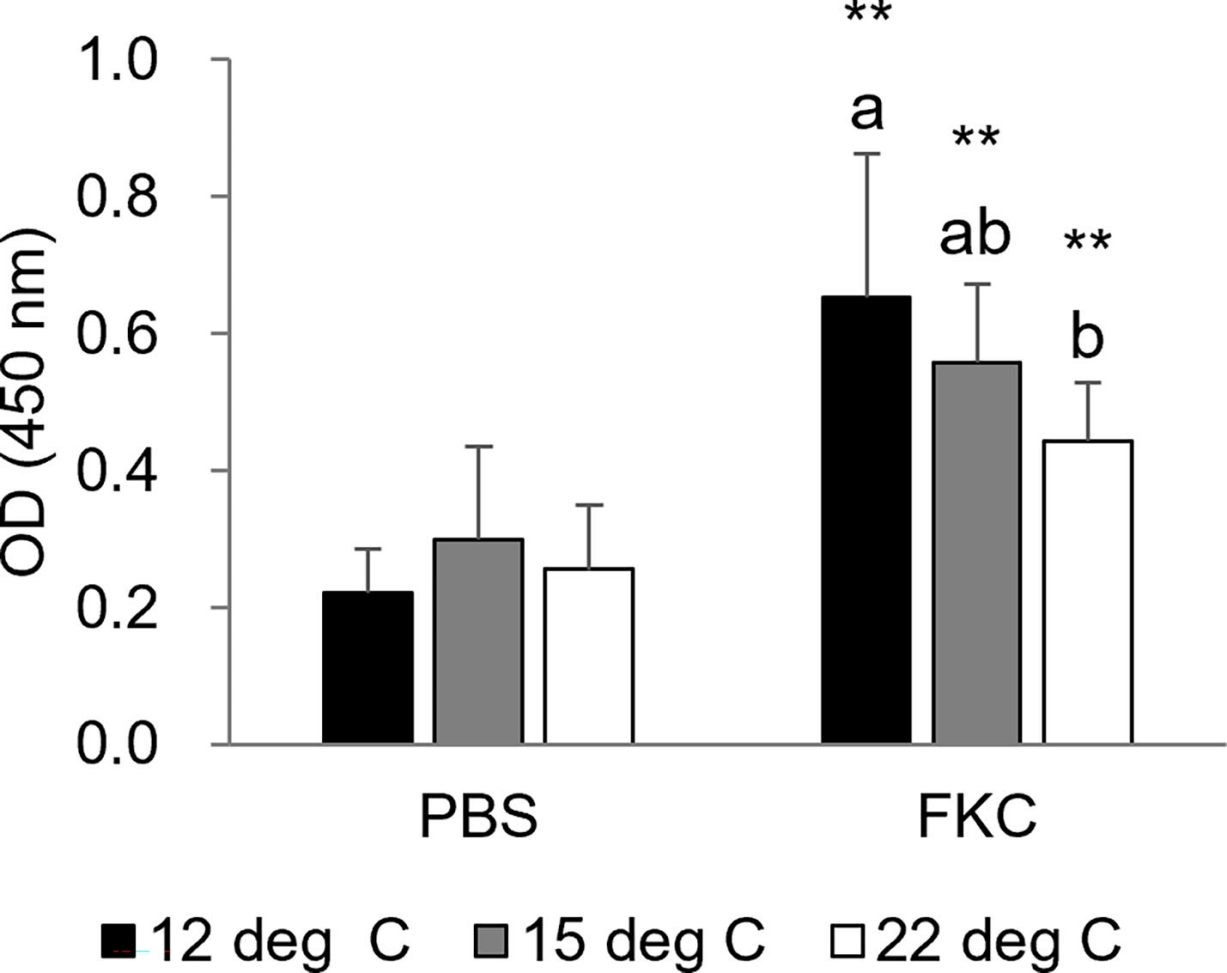
Serum antibody titer specific for *Flavobacterium psychrophilum* at 3 weeks after vaccination (*n* = 5-10). The black bars, gray bars and white bars indicate the 12°C group, 15°C group and 22°C group, respectively. Different characters indicate significant differences among the three groups in the same treatment as detected by ANOVA and the *post hoc* test. Asterisks indicate significant differences between PBS-injected and FKC-vaccinated fish in the same rearing water temperature group as detected by Student’s t test (** *P* < 0.01).

Similar to the results of the challenge test shown above, the cumulative survival rate of the challenged fish was relatively high in the 12°C group, medium in the 15°C group and low in the 22°C group (**Figures 6A-C**). Significant differences in the survival rate between FKC-vaccinated and PBS-injected fish were detected in all experimental groups, while the highest RPS value (34.8%) was observed in the 12°C group (**Table 3**). The RPS values tended to decrease in the 15°C group and 22°C group. Consistently, the antibody titer was higher in FKC-vaccinated fish of the 12°C group than in those of the 22°C group (**Figure 7**).

**Table 3:** Survival rate and relative percent survival of the vaccinated fish after bacterial challenge.

Group	Vaccination	Survival rate (survived/total)	RPS	Log-rank test
12°C	FKC	58.1% (18/31)	34.8	*p* = 6.111e-08
	PBS	35.7% (10/28)	–	–
15°C	FKC	46.7% (14/30)	15.8	*p* = 1.089e-07
	PBS	36.7% (11/30)	–	–
22°C	FKC	23.3% (7/30)	17.9	*p* = 2.031e-14
	PBS	6.7% (2/30)	–	–

## 4 Discussion

As most fish species are poikilotherms, their physiological functions, such as their immune system functions, are strongly affected by environmental water temperature. Ayu larvae produced under elevated water temperatures are usually said to exhibit increased susceptibility to infectious diseases among aquaculture farmers. Miwa et al. (12) and Hara et al. (13) reported that ayu reared at high water temperature lack a well-developed thymus, which has an important role in producing mature T cells. A similar phenomenon has also been observed in Japanese smelt, *Hypomesus nipponensis* (22). In this study, we showed that disease resistance to BCWD was significantly decreased in the 22°C group and tended to be decreased even in the 15°C group compared with the group of ayu reared at 12°C. In addition to the immune system, water temperature also affects the metabolic system of fish. The standard metabolic rate, which is required to maintain life and routine activity, usually increases with water temperature in poikilothermic fish species (5). The conversion efficiency of cultured fish is most efficient at suitable temperatures, while the efficiency decreases at higher temperatures (23). In this study, ayu reared at 22°C showed lower body lengths and weights than ayu in the other groups, showing lower conversion efficiency. Taken together, the findings indicate that improvement of the current protocol of ayu production is needed, especially in terms of water temperature during the juvenile-to-fry period, considering that temperature affects not only growth but also disease resistance.

Macrophage infiltration into the adipose tissue, which exhibits a crown-like structure, is a typical histopathological feature of adipose tissue in obese individuals (24). Free fatty acids from the enlarged adipocytes in obesity are recognized by infiltrated macrophages, resulting in the secretion of proinflammatory cytokines such as IL-6, TNFα and MCP-1 (25). Macrophages also surround dead adipocytes and secrete proinflammatory cytokines that induce local and systemic inflammation in obesity (26, 27). This chronic inflammation induced by danger signals, including fatty acids, was coined “homeostatic inflammation” by Maru, (28) and is thought to exacerbate allergies, cancers, and bacterial and viral infections (29, 30). In this study, macrophage infiltration, which produced structures similar to mammalian crown-like structures in adipose tissue, was found in ayu reared at 22°C. Increases in the numbers of melano-macrophages and upregulation of inflammatory cytokine genes such as IL-1β, IL-8 and TNFα were also observed in the spleens of ayu reared at 22°C. Furthermore, ayu reared at 22°C showed lower disease resistance to BCWD than those reared at 12°C. Thus, changes in the metabolic system caused by rearing at high water temperature may result in immune responses similar to mammalian homeostatic inflammation in the ayu, a poikilothermic fish species.

The thymus is a primary lymphoid organ that produces CD4+ and CD8+ T lymphocytes in vertebrates. Generally, the thymus shrinks and regresses with age after puberty, resulting in a decrease in naïve T-cell supply in mammals (31, 32). A correlation has been found between thymic size and T lymphocyte counts: infants with a larger thymic size have higher percentages of CD4- and CD8-positive lymphocytes than those with a smaller thymic size (33). Homeostatic proliferation maintains a constant level of T lymphocytes during lymphocytopenia in adulthood after thymic involution or in thymectomized young adults (34). Kato et al. (35) showed that the size of a population of a special phenotype of T lymphocytes, C-X-C chemokine receptor 3+ CD8+ naïve T cells, is increased in thymectomized mice and that this cell population enhances inflammatory responses producing IFN-γ and TNFα (35). In the present study, thymic involution was observed in ayu reared at elevated water temperatures, but the gene expression levels of CD4 and CD8 were comparable among the three groups. These data suggest that the mechanisms underlying the maintenance of T-cell numbers in thymic involution are also preserved in teleost fish. Furthermore, homeostatic proliferation of T cells, including the Treg subset, results in limited responsiveness to influenza vaccination in elderly people (36). The limited diversity of the CD4 T-cell repertoire in aged individuals, probably a consequence of age-related thymic involution, has been found to contribute to the poor response to influenza vaccination in a mouse model (37). In this study, vaccine efficacy and antibody titers gradually decreased along with the thymus size of ayu reared at different temperatures. Thymic involution induced by high water temperature may cause decreases in vaccine efficacy and antibody titers.

In conclusion, ayu reared at high water temperature (22°C) showed thymic involution, high condition factors, crown-like structure in adipose tissue, accumulation of melanin pigments in the spleen, high expression levels of inflammatory cytokine genes, and decreased resistance to BCWD. The data suggest that rearing at high water temperature induces metabolic syndrome-like features and thymic involution in ayu, resulting in a decrease in disease resistance and a poor response to vaccination. Although further studies are needed to show the details of the mechanisms underlying the decreased disease resistance, excessive inflammatory responses and poor response to vaccination, the present study provides insight into the physiological and immunological changes of fish under global warming.

## Data availability statement

The raw data supporting the conclusions of this article will be made available by the authors, without undue reservation.

## Ethics statement

The animal study was reviewed and approved by the Committee of Animal Experiments at Tokyo University of Marine Science and Technology (TUMSAT).

## Author contributions

GK and MS designed the study and wrote the manuscript. GK and HN performed histology, immunohistochemistry and gene expression analyses. KS and YK produced fish larvae and reared them in different water temperatures. HN, KS and YK performed vaccination and challenge tests. CN performed the ELISA experiment. KT and OK performed CT analyses.

## Supplementary material

Supplementary Figure 1 Schedules of fish rearing and sampling in Experiment 1 **(A)** and Experiment 2 **(B)**. GEA, gene expression analysis; ELISA, enzyme-linked immunosorbent assay.

Supplementary Figure 2 Fluorescence immunohistochemistry for *Flavobacterium psychrophilum* in the spleens of uninfected ayu reared at 12°C **(A)**, 15°C **(B)** and 22°C **(C)**. Data are representative of three individuals in each group.
