# Supplementary material for: Decreased resistance to bacterial cold-water disease and excessive inflammatory response in ayu (Plecoglossus altivelis) reared at high water temperature
Source: Front Immunol. 2023 Feb 2;14:1101491. doi: 10.3389/fimmu.2023.1101491 (PMC9931725; doi:10.3389/fimmu.2023.1101491)
Supplement: Supplementary file 1 [file Table_1.docx]

Supplementary Table 1 Primers used in this study.

| Primer name | Sequence | Accession No. |
| --- | --- | --- |
| CD4-1 F  CD4-1 R  CD8α F  CD8α R  IL-1β F  IL-1β R  IL-8 F  IL-8 R  IL-10 F  IL-10 R  TNFα F  TNFα R  EF-1α F  EF-1α R | 5´- GTCTGACTGTGAGGAACGTCA -3´  5´- CTACCACCACCCAGACGC -3´  5´- GGCCCCACATCAAAGGATCGC -3´  5´- ACTACCAAGTCGTGCGCCCC -3´  5´- TACCGGTTGGTACATCAGCA -3´  5´- TGACGGTAAAGTTGGTGCAA -3´  5´- GGAGCTGACCTTCGCTGCCA -3´  5´- TGGCCTGTCTGCTTCAGCGT -3´  5´- AGGTCTGTACAAAGCCATGGG -3´  5´- TTTAGATGCCAGGTACGTCTCG -3´  5´- CAAGGCTTCTCTGTCGGTAACC -3´  5´- CCCCTGCTTTTGATAACGATCT -3´  5´- GCTGCCGGCTCCTTCAC -3´  5´- AGATCTGTCCAGGGTGGTTCA -3´ | LC731293  LC535225  HF543937  KJ652902  KY799108  DD019003  EY510389 |
