# Supplementary material for: Decreased resistance to bacterial cold-water disease and excessive inflammatory response in ayu (Plecoglossus altivelis) reared at high water temperature
Source: Front Immunol. 2023 Feb 2;14:1101491. doi: 10.3389/fimmu.2023.1101491 (PMC9931725; doi:10.3389/fimmu.2023.1101491)
Supplement: Supplementary Figure 1 — Schedules of fish rearing and sampling in Experiment 1 (A) and Experiment 2 (B). GEA, gene expression analysis; ELISA, enzyme-linked immunosorbent assay. [file Presentation_1.pptx]

## Slide 1
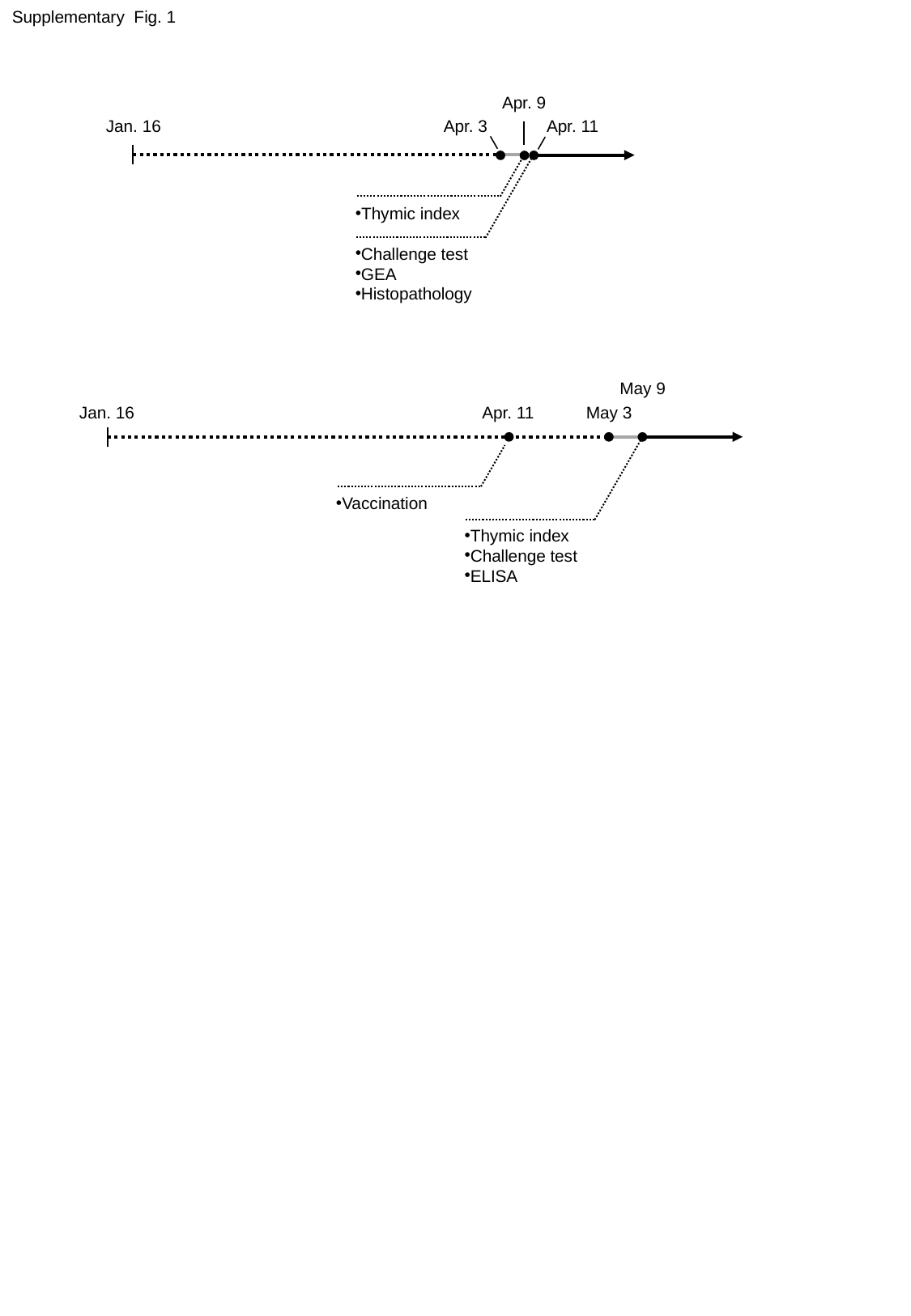

Supplementary Fig. 1
Apr. 9
Apr. 11
Jan. 16
Apr. 3
Thymic index
Challenge test
GEA
Histopathology
May 9
Jan. 16
May 3
Apr. 11
Vaccination
Thymic index
Challenge test
ELISA

## Slide 2
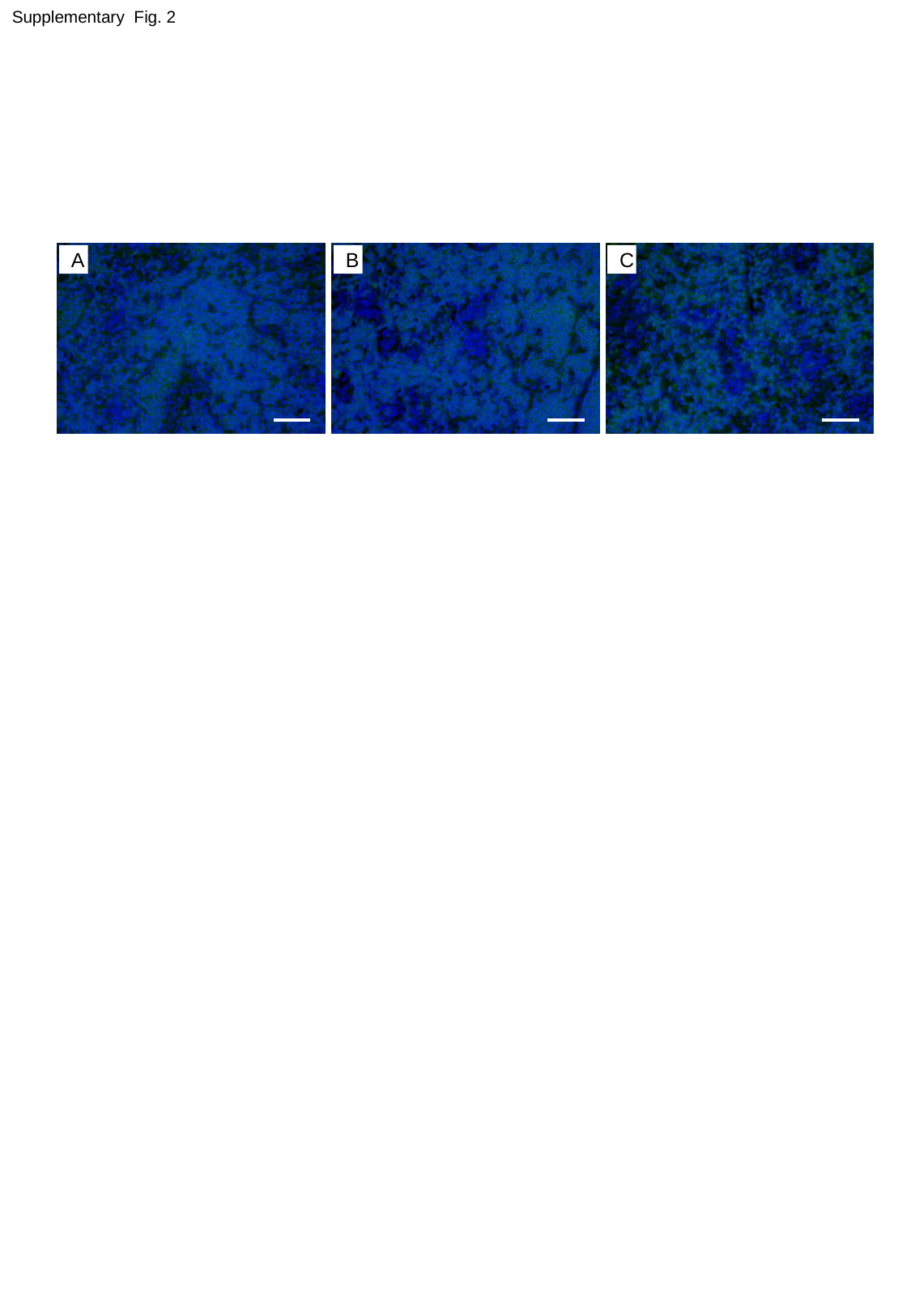

Supplementary Fig. 2
B
A
C
